# Component HCF Research Based on the Theory of Critical Distance and a Relative Stress Gradient Modification

**DOI:** 10.1371/journal.pone.0167722

**Published:** 2016-12-30

**Authors:** Songsong Sun, Xiaoli Yu, Zhentao Liu, Xiaoping Chen

**Affiliations:** 1 Power Machinery & Vehicular Engineering Institute, Zhejiang University, Hangzhou, China; 2 School of Mechanical Engineering, Ningbo University of Technology, Ningbo, China; Beihang University, CHINA

## Abstract

For the critical engine parts such as the crankshaft, the fatigue limit load is one of the most important parameters involved the design and manufacture stage. In previous engineering applications, this parameter has always been obtained by experiment, which is expensive and time-consuming. This paper, based on the theory of critical distance (TCD), first analyzes the stress distribution of a crankshaft under its limit load. In this way, the length of the critical distance can be obtained. Then a certain load is applied to a new crankshaft made of the same material and the effective stress is calculated based on the critical distance above. Finally, the fatigue limit load of the new crankshaft can be obtained by comparing the effective stress and the fatigue limit of the material. Comparison between the prediction and the corresponding experimental data shows that the traditional TCD may result in bigger errors on some occasions, while the modified TCD proposed in this paper can provide a more satisfactory result in terms of the fatigue limit for a quick engineering prediction.

## 1. Introduction

For the critical engineering parts such as the crankshaft, the important positions such as the fillet and the oil hole always have the property of variable cross-sections, which will result in the concentration of stress and the accumulated damage during the working period will be the main contributor to the final failure e.g., [1]. Thus, the correct prediction of the fatigue limit load of these parts becomes important, especially in the design stage before manufacturing. In previous work, excellent progress has been achieved in determining this parameter, and most of previous studies focus on the experiment and the analysis of the data e.g., [2]. This technique is easy to apply in the relatively simple structures such as the notched bars, while for a structure with a complicated shape; such as a crankshaft, there is still some difficulty in the engineering application. In addition, other factors such as the surface treatment technique, the residual stress, the manufacturing defects and the stress concentration factor will also have an outstanding impact on the fatigue strength and will result in some error in the prediction.

To solve this dilemma, creative work has been carried out in recent years. Among these methods, the theory of critical distance (TCD) is considered to be an effective method in component fatigue failure research. This approach was first proposed by Neuber and Peterson e.g., [3–4]; since then, Taylor has made significant contributions to its application e.g., [5–10]. This theory considers that the fatigue life of a given component not only depends on the maximum stress (which always occurs on the surface of the component), but is also influenced by the stress distribution within the vicinity of the stress concentration point. Based on this theory, Taylor predicted the fatigue life of some simple notched or welded components with good results. Later, Spaggiari compared this method with the classical stress gradient method and pointed out that some of the material parameters can obtained indirectly by this method e.g., [11]. Sun SS applied this method to the fatigue limit load prediction for various types of crankshafts and obtained a good result, but this work ignored the influence of the surface treatment on fatigue strength e.g., [12]. Benedetti combined the critical distance theory with a multiaxial fatigue criterion for predicting the fatigue strength of notched and plain shot-peened parts, this approach can predict the fatigue life of notched parts with residual stress e.g., [13]. Nicholas Gates also applied this approach with a multiaxial fatigue criterion and took other factors such as the stress gradient, notch root radius into account. Validation between the prediction and experimental data showed that compared with the traditional notch fatigue theory, this approach can get a much better accuracy in actual application e.g., [14].

In the traditional application of the TCD, the length of the critical distance is always considered to be a constant that depends solely on the properties of the material. In recent years, some experts have found that for a given component, the length of its critical distance depended not only on the properties of the material, but was also influenced by the structure. Because of this factor, several corresponding compensating methods have been proposed to modify the length of the critical distance. Hitherto, most of these compensating methods are based on the stress concentration factor e.g., [15–16]. However, for a geometrically complicated object such as a crankshaft, the stress concentration factor is difficult to measure either directly or indirectly. In addition, a crankshaft always has a more complex stress-strain state under an external applied load, as well as the property of multi-axial fatigue. Therefore, choosing a suitable kind of stress distribution to analyse is also an important matter.

This paper, based on this assumption, discusses the choice of the first proper failure criteria and the corresponding stress distributions are fitted by the finite element and the cubic interpolation method. Then, the value of the critical distance of a given crankshaft is obtained by analysing its stress distribution under the limit load. Finally, a new modification method based on the relative stress gradient is proposed to determine the length of the critical distance of the new crankshaft to predict the fatigue limit load. Comparison between the prediction and the experimental results shows that compared to the traditional TCD, this new modified approach may provide a more satisfactory result in terms of fatigue limit for quick engineering prediction.

## 2. Method

For the TCD applied in engineering work, the definition of the critical distance can be divided into two types: the direct definition and the indirect definition. The direct definition was proposed by Taylor D e.g., [5]. According to this definition, the length of this critical distance is:

$$L=\frac{1}{\pi }{\left(\frac{\Delta K_{th}}{\sigma_{b}}\right)}^{2}$$ (2.1)

In this definition, Δ*K*_*th*_ is the threshold value of the crack opening stress intensity factor, while *σ*_*b*_ is the limit of plain fatigue. The effective stress of the critical point method is:

$$\sigma_{eq}(PM)=\sigma (r=\frac{L}{2})$$ (2.2)

In this equation, *σ*_*eq*_(*PM*) is the effective stress obtained by the critical point method, and *r* is the distance from the critical point to the maximum stress point. For the critical line method, the effective stress is:

$$\sigma_{eq}(LM)=\frac{1}{2L}\int_{0}^{2L}\sigma (r)dr$$ (2.3)

From the definition above, we find that for a given component, the length of its critical distance according to this direct definition is just dependent on two parameters: the threshold value of the crack opening stress intensity factor and the limit of plain fatigue. In engineering application, these two parameters can be considered just depended on the material property. So for the components made by the same material, the values of the critical distance will be a constant.

The indirect definition of the critical distance was proposed by Neuber and Peterson, the expression according to this definition is^[^^3^^–^^4^^]^:

$$\sigma (r=L_{PM})=\sigma_{b}$$ (2.4)

$$\sigma (x=L_{LM})=\frac{1}{LM}\int_{0}^{LM}\sigma (x)dx=\sigma_{b}$$ (2.5)

In the two equations above, the subscripts LM and PM stand for the critical distance according to the line and point methods, respectively. For a given component, these two parameters can be obtained by analysing its stress distribution in the limit state. In previous engineering applications, these two parameters were all constants based on the property of the material. Therefore, after determining the length of the critical distance of a given crankshaft, the fatigue limit load of another crankshaft made of the same material but with a different structure could also be obtained. The process for this approach is as follows:

Step 1: Make the finite element analysis of a given crankshaft that has the bending fatigue test results. The critical load value is the limit load value of the crankshaft.
Step 2: Choose the suitable strength criteria and corresponding stress distribution of the crankshaft, then the length of the critical distance of this crankshaft can be determined by analysing the stress distribution.
Step 3: Apply a certain load on a new crankshaft of the same material but different structure and calculate the effective stress based on the same critical distance. Obtaining the limit load of the new crankshaft by comparing the limit effective stress and effective stress, the final fatigue limit load of the new crankshaft can be expressed as:
  $$M_{e}=\frac{\sigma_{b}}{\sigma_{eq}(A)}\times M_{A}$$ (2.6)
  where *M*_*e*_ and *M*_*A*_ are the prediction of the limit load and the certain load applied on the crankshaft, while *σ*_*b*_ and *σ*_*eq*_(*A*) are the fatigue limit of the material and the effective stress under the certain load *M*_*A*_ respectively.

**Fig 1:**
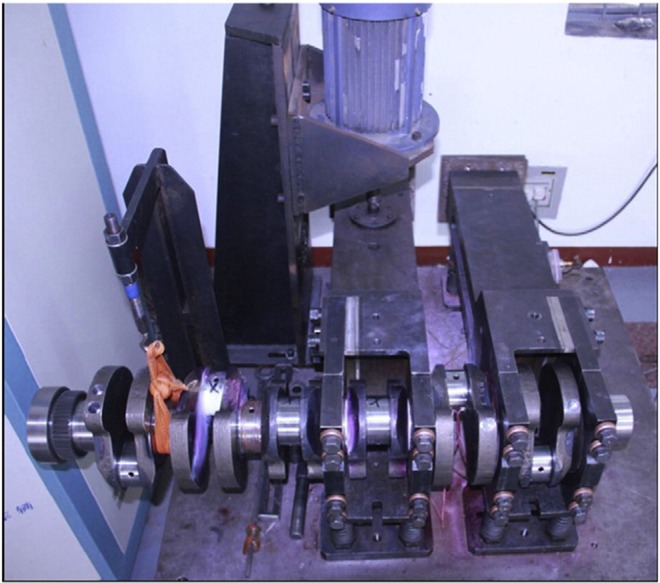
The experimental setup for the crankshaft.

To verify the accuracy of the prediction above, corresponding experimental verification is necessary. Fig 1 shows the experimental setup, which is composed of an electromagnetic vibration exciter, the master arm, the slave arm and the acceleration transducer. During the process of the experiment, the whole setup is fixed vertically on the foundation bed by several springs, and the excitation force is generated by rotating the eccentric wheel with the electromotor. In this way, a cyclic bending moment is applied on the crankshaft. As the experiment continues, the crack will appear at the fillet of the rod journal and the stiffness of the whole system decreases resulting in the increase of the acceleration if the frequency of the load remains unchanged. To avoid this unsatisfactory situation, the rotation speed of the electromotor will decrease accordingly. When the speed decreases by a certain value (in actual engineering application, this value is always specified to 60 rpm), the crankshaft is considered to be broken e.g., [17–18].

## 3. Results

### 3.1 Strength criteria selection

Compared with the simple structural notched components, the complicated shape of an engineering structure such as a crankshaft always has a more complex stress-strain state under external applied load; and exhibits the property of multi-axial fatigue. For actual engineering requirements, usually four strength criteria are considered: the first strength criteria (the maximum principal stress yield criteria), the second strength criteria (the maximum principal strain yield criteria), the third strength criteria (the maximum tangential stress criteria) and the fourth strength criteria (the Von Mises stress yield criteria).

In our study, the crankshaft is considered to be made of an elastic material, so the maximum principal stress is linearly related to the maximum principal strain. In addition, the failure model of the crankshaft is the shear failure model e.g., [19]. Of the four criteria above, only the third and fourth criteria take the shear stress into consideration with regard to the final fatigue, so these two critera are more suitable for the engineering application.

### 3.2 Critical distance based on the third strength criteria

The third strength criteria is the maximum tangential stress fatigue criteria, which suggests that the fatigue life of a given component is dependent on the maximum tangential stress. According to this criteria and TCD, the fatigue life of a certain component is influenced by the maximum tangential stress distribution within the vicinity of the stress concentration point.

According to the analysis in the previous chapter, the first step of this application is the determination of the length of the critical distance. Table 1 shows the test data for crankshaft No. 0. Chen et al. introduced the SAFL (Statistical Analysis for Fatigue Limit) theory to analyze fatigue test data e.g., [2], which indicate that the distribution of the limit load can be expressed by a normal distribution function. The independent variable in this function is the survival rate, while the dependent variable is the corresponding limit load. Using this function to fit the relationship between the survival rate (which can be replaced by the median rank in actual engineering application), the fatigue limit load of the crankshaft under the survival rate of 50% can be determined to be 5122.2 N∙m.

**Table 1:** Fatigue test data for crankshaft No. 0.

Load Moment(N·m)	Failure Serial Number	Median Rank
4786	1	0.067
4798	2	0.163
4877	3	0.260
5003	4	0.356
5015	5	0.452
5130	6	0.548
5131	7	0.644
5145	8	0.740
5233	9	0.837
5340	10	0.933

**Fig 2:**
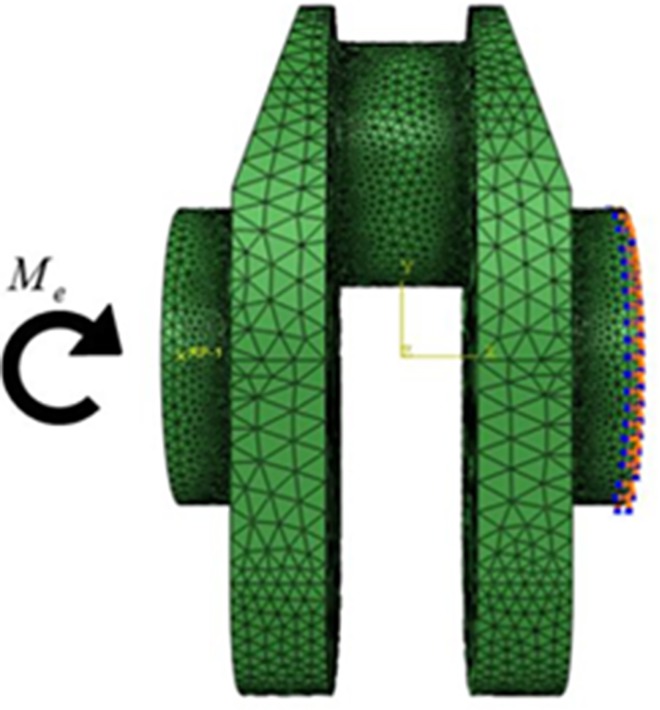
The FE(finite element)model of crankshaft No.0.

In this paper, the material of the crankshaft No. 0 is 42CrMo, and the surface treatment is nitridation. According to Gao et al e.g., [20], the strength factor of such a crankshaft is 1.3, so the fatigue limit load of the blank crankshaft is 3940 N∙m. Using this value in the finite element analysis, the finite element model is shown in Fig 2:

As shown, the bending moment is applied on the left face of the crankshaft, and the boundary condition is treated as restricting all the freedom on the right face. The number of the element in the model is 601777 and the type is A3D10. The details of the material are shown in Table 2.

**Table 2:** Material properties of the crankshaft.

Model Material Property	Value
Tensile strength	874 MPa
Yield strength	667 MPa
Young modulus	205000 MPa
Poisson’s ratio	0.29

As shown in Table 2, the tensile strength and the yield strength of the material are 874 MPa and 667 MPa respectively. Using the fatigue analysis software Femfat (Engineering Center Steyr GmbH & Co KG Steyrer Strasse 324300 Sankt Valentin, Austria) to estimate the fatigue limit of this material, the values of the plane fatigue limit and shear fatigue limit are estimated to be 392 MPa and 226 MPa respectively. Using the finite element method to obtain the maximum tangential stress distribution of crankshaft No. 0 under its fatigue limit load, the result is shown in Table 3, and the maximum value of the stress is 257.5 MPa.

**Table 3:** The maximum tangential stress distribution of the crank shaft No. 0(under its limit load).

Node Number	Distance (mm)	Stress (MPa)
1	0.1999	224.51
2	0.3998	199.61
3	0.5997	181.53
4	0.7996	163.72
5	0.9995	148.93
6	1.1994	134.93

**Fig 3:**
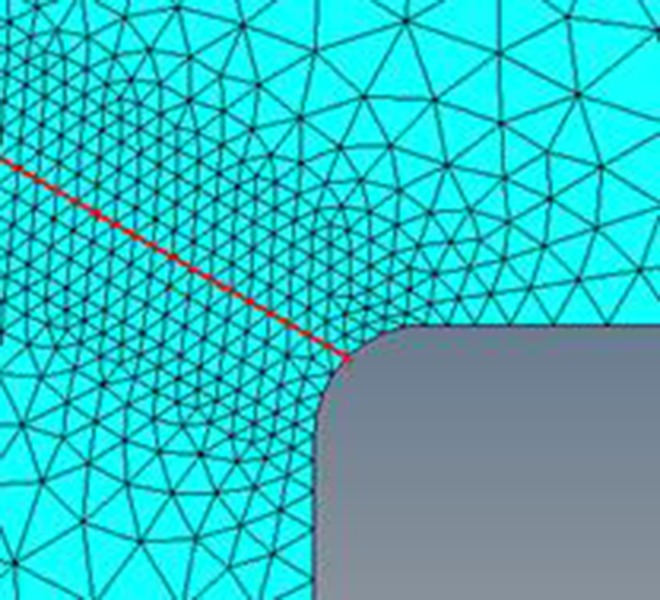
Positions of the nodes of crankshaft No.0.

In previous work, the stress distribution of the component was usually obtained by the finite element method, which exhibits a good efficiency in determining the values of the stress at the certain node (as showed in the red line in Fig 3). However, for the nodes of the element nodes, this approach will become a bit incorrect.

To solve this dilemma, some researchers proposed to fit the distribution curve using an interpolation method, where the independent variable is selected as the value of the distance, while the dependent variable is the value of the stress. Using this approach to fit the stress distribution, the result is:

$$\sigma (r=0.188)=226MPa=\sigma_{b}$$ (3.1)

**Fig 4:**
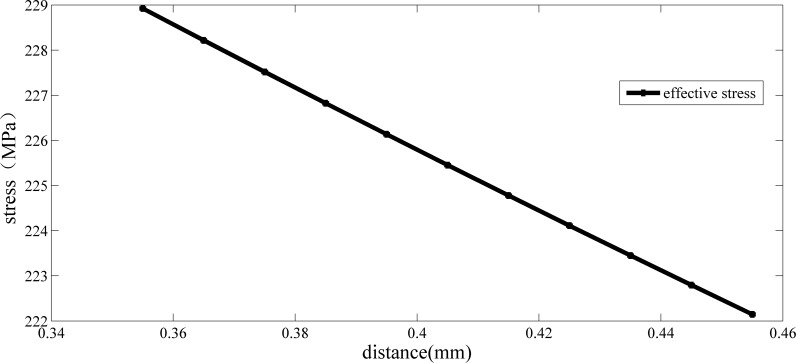
Relationship between the equivalent stress and critical distance of crankshaft No.0 (under its limit load and the third strength criteria).

According to the definition of TCD, the length of the critical distance based on this strength criteria and critical point method is 0.188 mm. Calculating the effective stress of this crankshaft at a different distance, the result is shown in Fig 4:

As shown in Fig 4, when the distance is 0.395 mm, the effective stress is 226 MPa. According to the definition of TCD, the length of the critical distance based on this strength criteria and critical line method is 0.395 mm. Applying a bending moment to a new crankshaft (No .1) made of the same material, the value of the load is 1000 N∙m. The maximum tangential stress distribution is shown in Table 4 and the maximum value of the stress is 118.5 MPa.

**Table 4:** The maximum tangential stress distribution of the crank shaft No. 1(under 1000 N∙m).

Node Number	Distance (mm)	Stress (MPa)
1	0.2048	95.4
2	0.4096	78.7
3	0.6144	68.7
4	0.8192	58.3
5	1.024	51.6
6	1.2288	45.1

The interpolation method was repeated to fit the stress distribution and calculate the effective stress according to both the critical point and the critical line method respectively. The results are:

σ(PM)=σ(r=0.188)=96.98MPa (3.2)

$$\sigma (LM)=\frac{1}{0.395}\int_{0}^{0.395}\sigma (x)dx=97.04MPa$$ (3.3)

By comparing the effective stress and the shear fatigue limit of the material, the fatigue limit load of crankshaft No. 1 is calculated to be:

$$M(PM)=\frac{226}{96.98}\times 1000=2330.4N\cdot m$$ (3.4)

$$M(LM)=\frac{226}{97.04}\times 1000=2328.4N\cdot m$$ (3.5)

### 3.3 Critical distance based on the fourth strength criteria

The fourth strength criteria is just the Von Mises stress yield criteria, which suggests that the failure state of a given component depends on the Von Mises equivalent stress. Thus, the corresponding conclusion can be reached that the fatigue life of a given component is influenced by the Von Mises stress distribution within the vicinity of the stress concentration point by combining this criteria and TCD.

Repeating the finite element method in the previous section to analyse the Von Mises stress distribution of crankshaft No. 0 under its limit load, the result is shown in Table 5, and the maximum value of the stress is 449.6 MPa.

**Table 5:** The Von Mises stress distribution of the crankshaft No. 0 (under its limit load).

Node Number	Distance (mm)	Stress (MPa)
1	0.1999	397.44
2	0.3998	359.99
3	0.5997	329.08
4	0.7996	298.55
5	0.9995	273.28
6	1.1994	249.46

When an interpolation method is used to fit the Von Mises stress distribution curve, the result is shown below:

$$\sigma (r=0.225)=392MPa=\sigma_{b}$$ (3.6)

**Fig 5:**
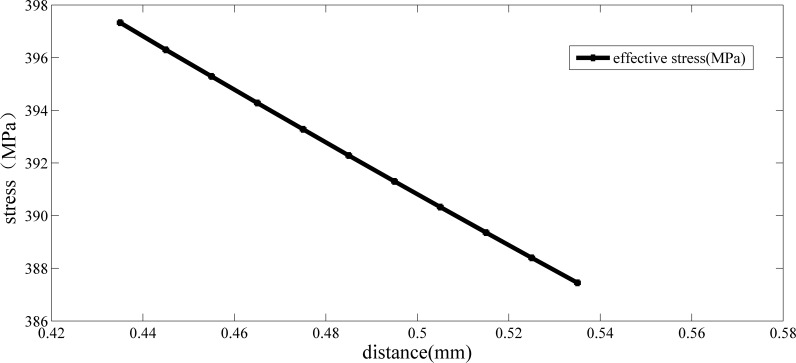
Relationship between the equivalent stress and critical distance of crankshaft No.0 (under its limit load and the fourth strength criteria).

As shown in Eq (3.6), the critical distance based on the fourth strength criteria and the critical point method is 0.225 mm. Calculating the effective stress of this crankshaft at a different distance, the result is shown in Fig 5:

As shown in Fig 5, when the distance is 0.485 mm, the effective stress is 392 MPa. According to the definition of TCD, the length of the critical distance based on this strength criteria and the critical line method is 0.485 mm. Applying a bending moment to crankshaft No. 1, the value of the load is 1000 N∙m. The maximum tangential stress distribution is shown in Table 6 and the maximum value of the stress is 210.7 MPa.

**Table 6:** The Von Mises stress distribution of the crankshaft No. 1 (under 1000 N∙m).

Node Number	Distance (mm)	Stress (MPa)
1	0.2048	171.353
2	0.4096	142.746
3	0.6144	125.758
4	0.8192	107.869
5	1.024	96.5998
6	1.2288	85.52

The interpolation method mentioned above was used to fit the stress distribution and calculate the effective stress according to both the critical point and the critical line method respectively. The results are:

σ(PM)=σ(r=0.225)=168.1MPa (3.7)

$$\sigma (LM)=\frac{1}{0.485}\int_{0}^{0.485}\sigma (x)dx=167.83MPa$$ (3.8)

By comparing the effective stress and the plan fatigue limit of the material, the fatigue limit load of crankshaft No. 1 is:

$$M(PM)=\frac{392}{168.1}\times 1000=2331.9N\cdot m$$ (3.9)

$$M(LM)=\frac{392}{167.83}\times 1000=2335.7N\cdot m$$ (3.10)

## 4. Experimental verification

As mentioned above, the fatigue limit load of a given crankshaft is always obtained by the bending fatigue test bench. Using this equipment to perform a fatigue test of crankshaft No. 1, the result of the experiment is shown in Table 7:

**Table 7:** Fatigue test data for crankshaft No. 1.

Load Moment(N·m)	Failure Serial Number	Median Rank
2796	1	0.067
3213	2	0.163
3263	3	0.260
3331	4	0.356
3353	5	0.452
3377	6	0.548
3379	7	0.644
3481	8	0.740
3757	9	0.837
3779	10	0.933

By using the same calculation method mentioned above, the fatigue limit load of this crankshaft can be determined to be 3335 N∙m. Then, the fatigue limit of the blank crankshaft is 2565.5 N∙m. Comparing the data and the prediction, the corresponding errors are in Table 8:

**Table 8:** Prediction error based on different strength criteria and conventional TCD.

Third Strength Criteria		Fourth Strength Criteria	
Critical method	Error	Critical method	Error
Point	9.2%	Point	9.1%
Line	9.3%	Line	8.9%

As shown in Table 8, the prediction based on the conventional TCD and the different strength criteria may result in some errors (over 5%), mainly because of the structural difference between these two crankshafts. For crankshaft No. 0, the fillet radius of its main journal is equal to 5 mm. While for crankshaft No. 1, this parameter is 3 mm. According to Chen et al e.g., [21], among the main structural parameters of the crankshaft, the fillet radius of the main journal influences the fatigue strength significantly.

## 5. Modification of the critical distance

### 5.1 Modification based on the relative stress gradient

From the analysis above, a primary conclusion can be drawn that for a given component, when the conventional TCD is applied to its fatigue limit prediction, the value of the critical distance is not only dependent on the material, but is also influenced by the structure. Thus, a quick determination of the critical distance for a given component has a realistic engineering meaning, especially in the design stage.

To solve this dilemma, pioneering attempts have been conducted in recent years and several corresponding modification methods are proposed. Up to now, most of these modification techniques are based on the stress concentration factor. For the simple structural notched components, this parameter is very easy to obtain. However, for the crankshaft, there is still no standard definition of this parameter in actual engineering application.

In previous work, some researchers noted that the stress gradient is an effective parameter in evaluating the size effect of the component e.g., [12–14]. Based on this assumption, this paper proposes a new modification method with the following expression:

$$L_{PM}=c\cdot M$$ (5.1)

$$L_{LM}=c\cdot N$$ (5.2)

where both *M* and *N* are parameters based on the properties of the material, while *c* is the relative stress gradient at the maximum stress point. The relevant expression is shown below:

$$c=\frac{1}{\sigma (x)}\frac{d(\sigma (x))}{dx}|x=0$$ (5.3)

### 5.2 Stress gradient calculation based on an extrapolation method

For most of the crankshafts, the stress distributions under the corresponding limit load can not be fitted directly by a simple curve, so the stress gradient can not be calculated directly either. To solve this problem, some researchers proposed that the stress gradient at the maximum stress point can be obtained indirectly by a combined finite element and extrapolation method e.g., [12]. The first step of this approach is to calculate the stress gradient at every node of the element, and the result is shown below:

$$s_{i}=\frac{\sigma (0)-\sigma (x_{i})}{r_{i}}$$ (5.4)

where *s*_*i*_ is the stress gradient at node *i*, *σ*(*x*_*i*_) is the value of the stress, and *r*_*i*_ is the distance between this node and the maximum stress point. According to this definition, the stress gradient distribution of crankshaft No. 0 based on different strength criteria can be calculated.

**Fig 6:**
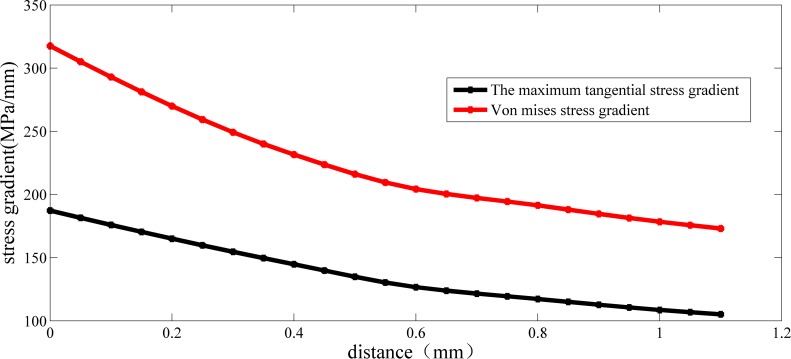
Stress gradient distribution of crankshaft No.0(under its limit load).

As shown in Table 9, the stress gradient at every node can be computed directly. Therefore, for the stress gradient at the maximum stress point can be obtained indirectly by an extrapolation method, where the independent variable is selected as the value of the distance, while the dependent variable is the value of the stress gradient at these nodes. Using this approach to obtain the stress gradient at the maximum stress point, the results are in Fig 6.

**Table 9:** Stress gradient distribution for crankshaft No. 0 (under its limit load).

Node Number	Distance (mm)	Maximum Tangential Stress Gradient (MPa/mm)	Von Mises Stress Gradient(MPa/mm)
1	0.1999	165.0325163	260.9304652
2	0.3998	144.7973987	224.1370685
3	0.5997	126.6800067	200.9671502
4	0.7996	117.2836418	188.9069535
5	0.9995	108.6243122	176.4082041
6	1.1994	102.192763	166.8667667

As shown in Fig 6, when crankshaft No. 0 is under its limit load, the maximum tangential stress gradient and the Von Mises stress gradient are 187.27 MPa/mm and 308.25 MPa/mm, respectively. In the previous section, the maximum values of the maximum tangential stress and Von Mises stress are 257.5 MPa and 449.6 MPa, respectively. According to the definition, the relative stress gradient based on these two strength criteria are 0.728 mm^-1^ and 0.686 mm^-1^. Combining these two values and the corresponding critical distance of crankshaft No. 0, the values of both both *M* and *N* based on the third strength can be determined to be:

$$M=\frac{L_{PM}}{c}=\frac{0.188}{0.728}=0.258mm^{2}/MPa$$ (5.5)

$$N=\frac{L_{LM}}{c}=\frac{0.395}{0.728}=0.543mm^{2}/MPa$$ (5.6)

For the fourth strength criteria, the value of these two parameters are shown below:

$$M=\frac{L_{PM}}{c}=\frac{0.225}{0.686}=0.328mm^{2}/MPa$$ (5.7)

$$N=\frac{L_{LM}}{c}=\frac{0.485}{0.686}=0.707mm^{2}/MPa$$ (5.8)

When this extrapolation technique is repeated to obtain the relative stress gradient of crankshaft No. 1, the stress gradient of each node is shown in Table 10:

**Table 10:** Stress gradient distribution of crankshaft No. 1 (under 1000 N∙m).

Node number	Distance (mm)	Maximum Tangential Stress Gradient (MPa/mm)	Von Mises Stress Gradient(MPa/mm)
1	0.2048	113.2128906	193.3447266
2	0.4096	97.33764648	166.5136719
3	0.6144	81.0530599	138.6588542
4	0.8192	73.55957031	125.8312988
5	1.024	65.36523438	111.6701172
6	1.2288	59.7759196	102.0751953

**Fig 7:**
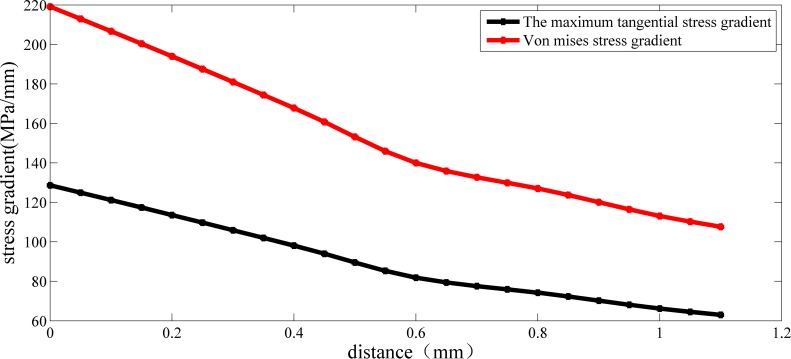
Stress gradient distribution of crankshaft No.1(under 1000 N∙m).

The extrapolation method was used to obtain the stress gradient at the maximum stress point, and the results are shown in Fig 7:

As shown in Fig 7, when crankshaft No. 1 is under 1000 N∙m, the maximum tangential stress gradient and the Von Mises stress gradient are 128.67 MPa/mm and 219.13 MPa/mm, respectively. The maximum value of the maximum tangential stress and Von Mises stress under this load are 118.5 MPa and 210.95 MPa, respectively. According to the definition, the relative stress gradients based on these two strength criteria are 1.086 mm^-1^ and 1.039 mm^-1^. Combining these parameters and the modification method above, the critical distance based on the third strength criteria is shown below:

$$L_{PM}=1.086\times 0.258=0.28mm$$ (5.9)

$$L_{LM}=1.086\times 0.543=0.59mm$$ (5.10)

For the fourth strength criteria, the value of the critical distance is shown below:

$$L_{PM}=1.039\times 0.328=0.34mm$$ (5.11)

$$L_{LM}=1.039\times 0.707=0.735mm$$ (5.12)

### 5.3 Prediction based on the modified critical distance

Based on the modified critical distance, the corresponding effective stress can be computed. For the third strength criteria, the results are calculated below:

σ(PM)=σ(r=0.28)=88.47MPa (5.13)

$$\sigma (LM)=\frac{1}{0.59}\int_{0}^{0.59}\sigma (x)dx=89.58MPa$$ (5.14)

By comparing the effective stress and the shear fatigue limit of the material, the fatigue limit load of crankshaft No. 1 under this strength criteria is shown below:

$$M(PM)=\frac{226}{88.47}\times 1000=2554.5N\cdot m$$ (5.15)

$$M(LM)=\frac{226}{89.58}\times 1000=2522.9N\cdot m$$ (5.16)

While for the fourth strength criteria, the effective stress is shown below:

σ(PM)=σ(r=0.34)=151.1MPa (5.17)

$$\sigma (LM)=\frac{1}{0.735}\int_{0}^{0.735}\sigma (x)dx=153.7MPa$$ (5.18)

Comparing the effective stress and the plan fatigue limit of the material, the fatigue limit load of crankshaft No. 1 under this strength criteria is shown below:

$$M(PM)=\frac{392}{151.1}\times 1000=2594.3N\cdot m$$ (5.19)

$$M(LM)=\frac{392}{153.7}\times 1000=2550.1N\cdot m$$ (5.20)

Comparing the prediction and the experimental data, the corresponding errors are shown in Table 11:

**Table 11:** Prediction error based on different strength criteria and modified TCD.

Third Strength Criteria		Fourth Strength Criteria	
Critical method	Error	Critical method	Error
Point	0.4%	Point	1.1%
Line	1.7%	Line	0.6%

As shown in Table 11, compared with the conventional TCD, the modified TCD can obtain a much better accuracy in predicting the fatigue limit load of crankshaft No. 1 based on both the maximum tangential stress and the Von Mises stress distribution (the errors are less than 5%), so this approach is more suitable for the actual engineering application.

## 6. Conclusion

Conventional TCD always considers the length of the critical distance to be a constant in actual engineering applications. This paper first chooses the third and fourth strength criteria and analyses the corresponding stress distribution of a crankshaft under its limit load. In this way, the critical distance of this crankshaft based on different strength criteria can be obtained indirectly. Then, the fatigue limit load of another crankshaft made of the same material is computed based on the critical distance previously obtained. Comparison between the predicted and experimental results shows that compared with the conventional TCD, the modified TCD can exhibit a better accuracy in fatigue limit prediction and is thus more suitable for engineering applications.

## Supporting Information

S1 Dataset Minimal data set. (DOCX)

S1 File Supplementary tables. (DOCX)
