# Supplementary material for: Component HCF Research Based on the Theory of Critical Distance and a Relative Stress Gradient Modification
Source: PLoS One. 2016 Dec 30;11(12):e0167722. doi: 10.1371/journal.pone.0167722 (PMC5201284; doi:10.1371/journal.pone.0167722)
Supplement: S1 Dataset — (DOCX) [file pone.0167722.s001.docx]

Fig list

Fig 1 The experimental setup for the crankshaft

Fig 2 The FE(finite element)model of crankshaft No.0

Fig 3 Positions of the nodes of crankshaft No.0

Fig 4 Relationship between the equivalent stress and critical distance of crankshaft No.0 (under its limit load and the third strength criteria)

Fig 5 Relationship between the equivalent stress and critical distance of crankshaft No.0 (under its limit load and the fourth strength criteria)

Fig 6 Stress gradient distribution of crankshaft No.0(under its limit load)

Fig 7 Stress gradient distribution of crankshaft No.1(under 1000 N∙m)


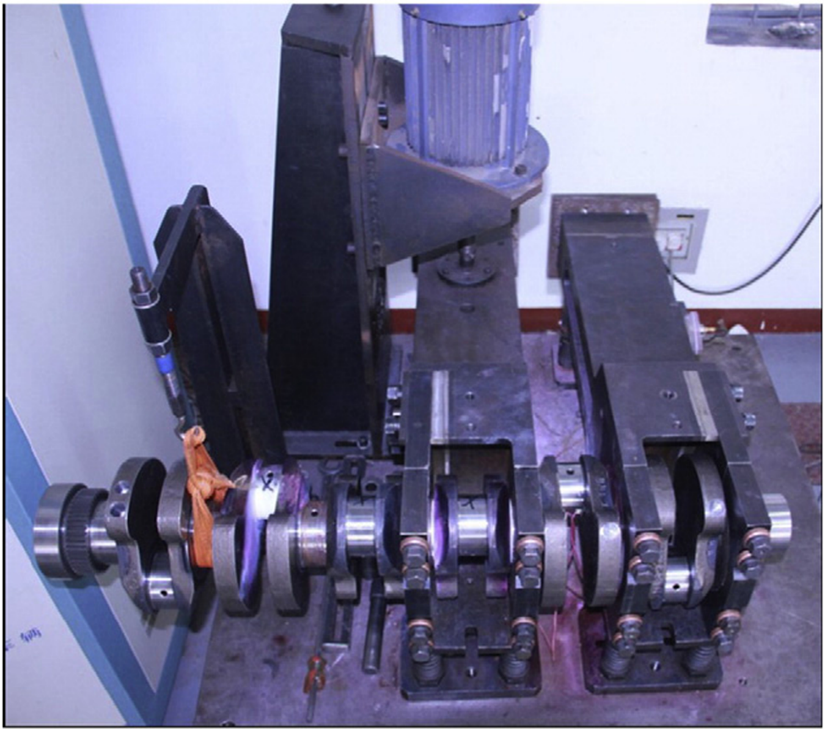


Fig 1 The experimental setup for the crankshaft


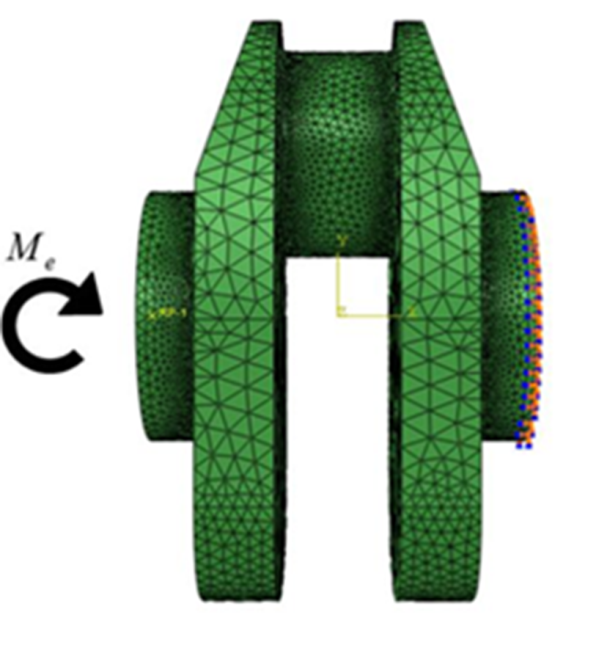


Fig 2 The FE(finite element)model of crankshaft No.0


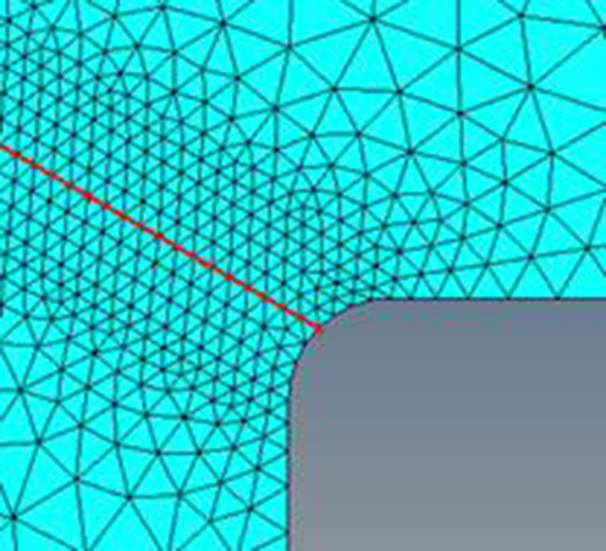


Fig 3 Positions of the nodes of crankshaft No.0


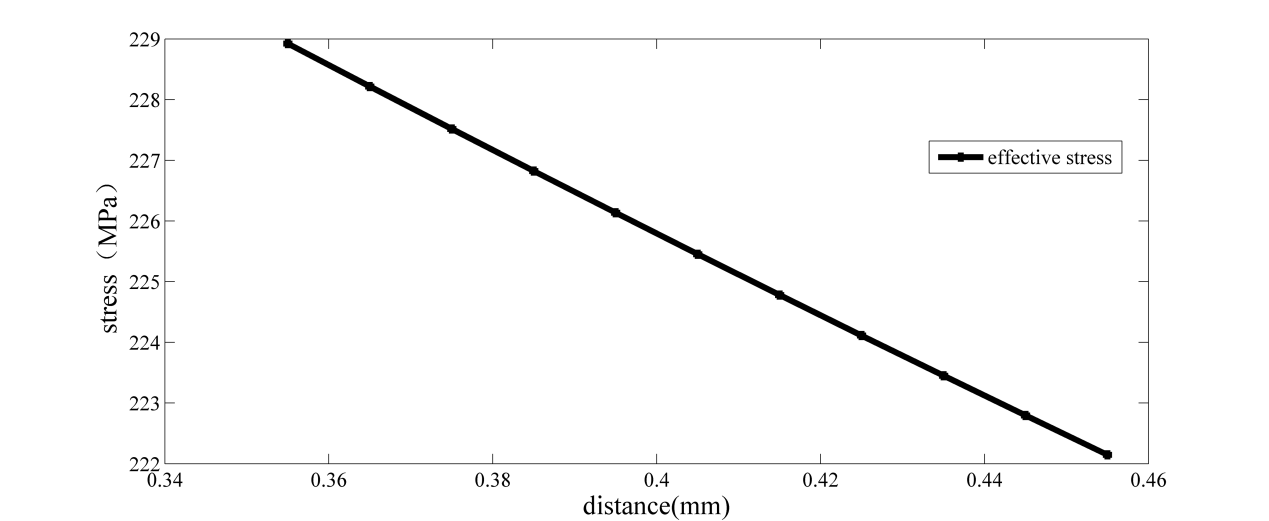


Fig 4 Relationship between the equivalent stress and critical distance of crankshaft No.0 (under its limit load and the third strength criteria)


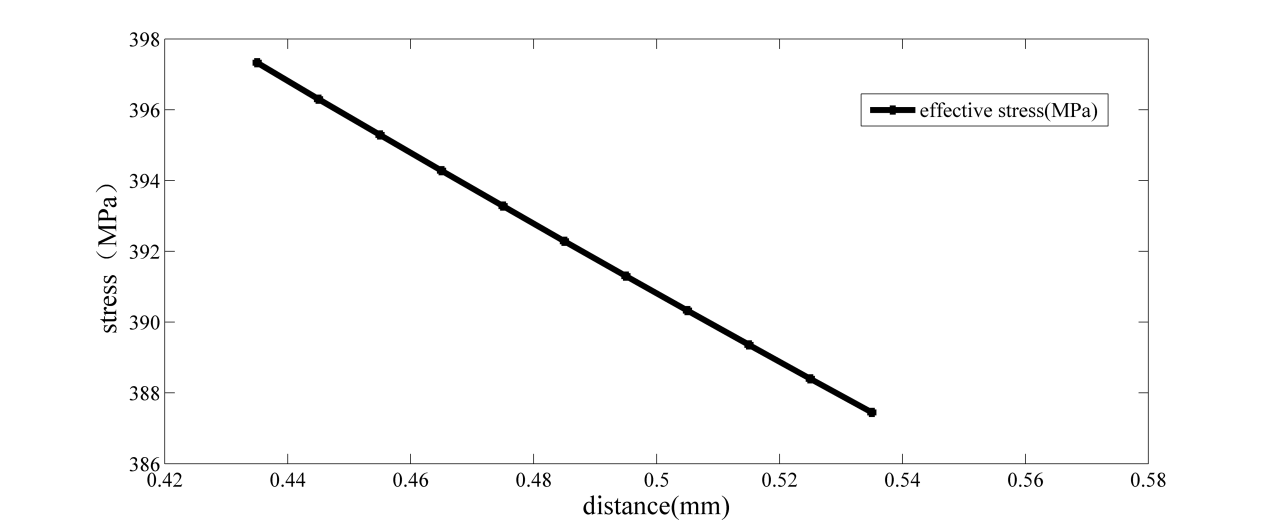


Fig 5 Relationship between the equivalent stress and critical distance of crankshaft No.0 (under its limit load and the fourth strength criteria)


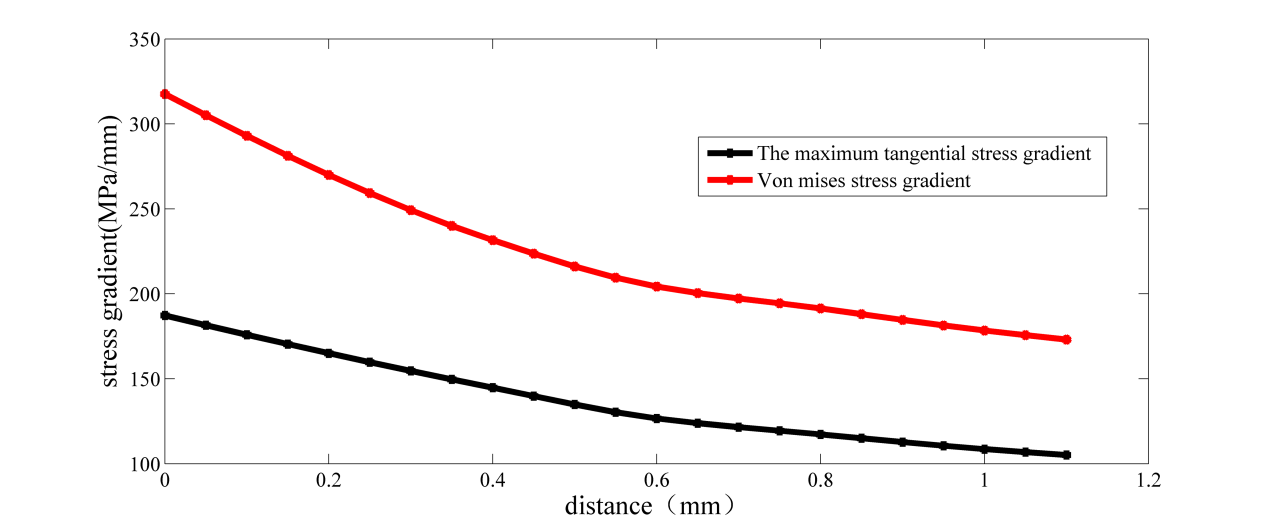


Fig 6 Stress gradient distribution of crankshaft No.0(under its limit load)


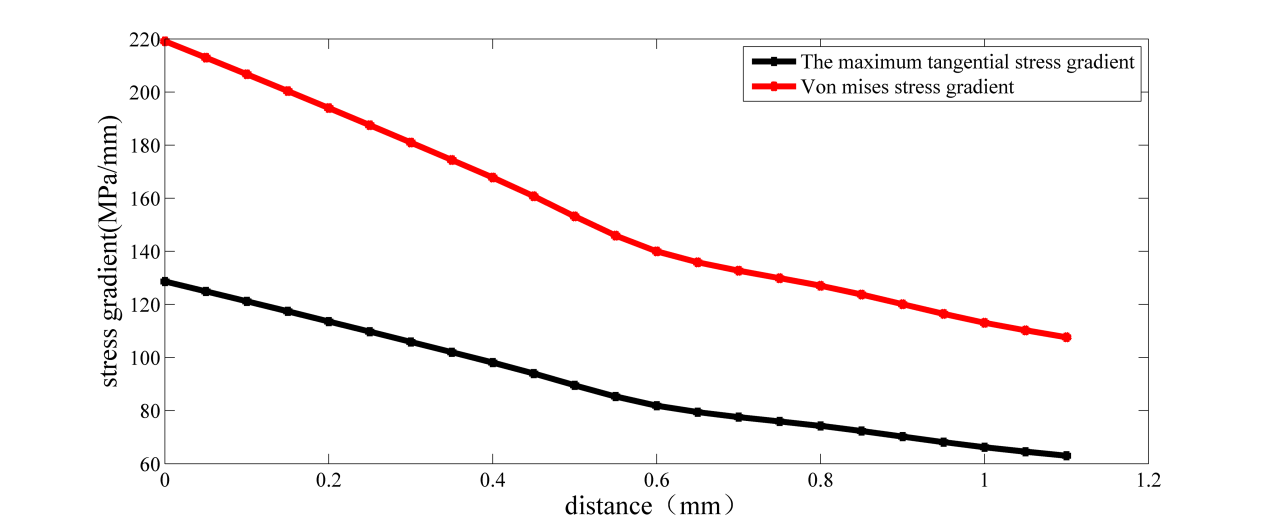


Fig 7 Stress gradient distribution of crankshaft No.1(under 1000 N∙m)

Table list

Table 1. Fatigue test data for crankshaft No. 0

Table 2. Material properties of the crankshaft

Table 3. The maximum tangential stress distribution of the crank shaft No. 0(under its limit load)

Table 4. The maximum tangential stress distribution of the crank shaft No. 1(under 1000 N∙m)

Table 5 The Von Mises stress distribution of the crankshaft No. 0 (under its limit load)

Table 6 The Von Mises stress distribution of the crankshaft No. 1 (under 1000 N∙m)

Table 7 Fatigue test data for crankshaft No. 1

Table 8 Prediction error based on different strength criteria and conventional TCD

Table 9 Stress gradient distribution for crankshaft No. 0 (under its limit load)

Table 10 Stress gradient distribution of crankshaft No. 1 (under 1000 N∙m)

Table 11 Prediction error based on different strength criteria and modified TCD

Table 1. Fatigue test data for crankshaft No. 0

| Load Moment(N·m) | Failure Serial Number | Median Rank |
| --- | --- | --- |
| 4786 | 1 | 0.067 |
| 4798 | 2 | 0.163 |
| 4877 | 3 | 0.260 |
| 5003 | 4 | 0.356 |
| 5015 | 5 | 0.452 |
| 5130 | 6 | 0.548 |
| 5131 | 7 | 0.644 |
| 5145 | 8 | 0.740 |
| 5233 | 9 | 0.837 |
| 5340 | 10 | 0.933 |

Table 2. Material properties of the crankshaft

| Model Material Property | Value |
| --- | --- |
| Tensile strength | 874 MPa |
| Yield strength | 667 MPa |
| Young modulus | 205000 MPa |
| Poisson’s ratio | 0.29 |

Table 3. The maximum tangential stress distribution of the crank shaft No. 0(under its limit load)

| Node Number | Distance (mm) | Stress (MPa) |
| --- | --- | --- |
| 1 | 0.1999 | 224.51 |
| 2 | 0.3998 | 199.61 |
| 3 | 0.5997 | 181.53 |
| 4 | 0.7996 | 163.72 |
| 5 | 0.9995 | 148.93 |
| 6 | 1.1994 | 134.93 |

Table 4. The maximum tangential stress distribution of the crank shaft No. 1(under 1000 N∙m)

| Node Number | Distance (mm) | Stress (MPa) |
| --- | --- | --- |
| 1 | 0.2048 | 95.4 |
| 2 | 0.4096 | 78.7 |
| 3 | 0.6144 | 68.7 |
| 4 | 0.8192 | 58.3 |
| 5 | 1.024 | 51.6 |
| 6 | 1.2288 | 45.1 |

Table 5 The Von Mises stress distribution of the crankshaft No. 0 (under its limit load)

| Node Number | Distance (mm) | Stress (MPa) |
| --- | --- | --- |
| 1 | 0.1999 | 397.44 |
| 2 | 0.3998 | 359.99 |
| 3 | 0.5997 | 329.08 |
| 4 | 0.7996 | 298.55 |
| 5 | 0.9995 | 273.28 |
| 6 | 1.1994 | 249.46 |

Table 6 The Von Mises stress distribution of the crankshaft No. 1 (under 1000 N∙m)

| Node Number | Distance (mm) | Stress (MPa) |
| --- | --- | --- |
| 1 | 0.2048 | 171.353 |
| 2 | 0.4096 | 142.746 |
| 3 | 0.6144 | 125.758 |
| 4 | 0.8192 | 107.869 |
| 5 | 1.024 | 96.5998 |
| 6 | 1.2288 | 85.52 |

Table 7 Fatigue test data for crankshaft No. 1

| Load Moment(N·m) | Failure Serial Number | Median Rank |
| --- | --- | --- |
| 2796 | 1 | 0.067 |
| 3213 | 2 | 0.163 |
| 3263 | 3 | 0.260 |
| 3331 | 4 | 0.356 |
| 3353 | 5 | 0.452 |
| 3377 | 6 | 0.548 |
| 3379 | 7 | 0.644 |
| 3481 | 8 | 0.740 |
| 3757 | 9 | 0.837 |
| 3779 | 10 | 0.933 |

Table 8 Prediction error based on different strength criteria and conventional TCD

| Third Strength Criteria | | Fourth Strength Criteria | |
| --- | --- | --- | --- |
| Critical method | Error | Critical method | Error |
| Point | 9.2% | Point | 9.1% |
| Line | 9.3% | Line | 8.9% |

Table 9 Stress gradient distribution for crankshaft No. 0 (under its limit load)

| Node Number | Distance (mm) | Maximum Tangential Stress Gradient (MPa/mm) | Von Mises Stress Gradient(MPa/mm) |
| --- | --- | --- | --- |
| 1 | 0.1999 | 165.0325163 | 260.9304652 |
| 2 | 0.3998 | 144.7973987 | 224.1370685 |
| 3 | 0.5997 | 126.6800067 | 200.9671502 |
| 4 | 0.7996 | 117.2836418 | 188.9069535 |
| 5 | 0.9995 | 108.6243122 | 176.4082041 |
| 6 | 1.1994 | 102.192763 | 166.8667667 |

Table 10 Stress gradient distribution of crankshaft No. 1 (under 1000 N∙m)

| Node number | Distance (mm) | Maximum Tangential Stress Gradient (MPa/mm) | Von Mises Stress Gradient(MPa/mm) |
| --- | --- | --- | --- |
| 1 | 0.2048 | 113.2128906 | 193.3447266 |
| 2 | 0.4096 | 97.33764648 | 166.5136719 |
| 3 | 0.6144 | 81.0530599 | 138.6588542 |
| 4 | 0.8192 | 73.55957031 | 125.8312988 |
| 5 | 1.024 | 65.36523438 | 111.6701172 |
| 6 | 1.2288 | 59.7759196 | 102.0751953 |

Table 11 Prediction error based on different strength criteria and modified TCD

| Third Strength Criteria | | Fourth Strength Criteria | |
| --- | --- | --- | --- |
| Critical method | Error | Critical method | Error |
| Point | 0.4% | Point | 1.1% |
| Line | 1.7% | Line | 0.6% |
